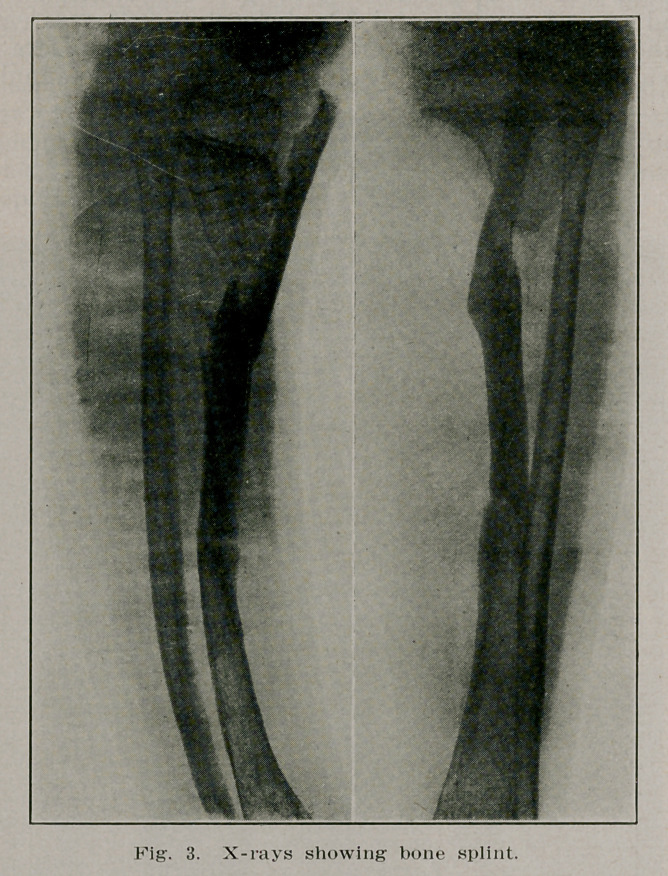# Report of an Unusual Case of Ununited Fracture of the Tibia, Repaired by Bone Grafting

**Published:** 1915-01

**Authors:** Prescott Le Breton

**Affiliations:** Buffalo, N. Y.


### Report of an Unusual Case of Ununited Fracture of the Tibia, Repaired by Bone Grafting.

By PRESCOTT LE BRETON, M. D.

Buffalo, N. Y.

The following case is reported, because union was obtained in spite of the fact that a long time had elapsed since the fracture (practically during the entire life of the patient). Also because no method of plating would have been satisfactory, owing to the atrophy of the bone ends, but bone grafting with plenty of bony contact was necessary to ensure union. The photographs and X-rays show the condition before and after operation.

Fig. 1.

E. R., aged seven, referred by Dr. McKee, June 4, 1913. When this boy was three weeks old, he sustained a fracture of the right tibia a little below the knee joint. Through some neglect the bandages and splints slipped soon after the dressing was applied and were not replaced. On beginning to walk, it was noticed that the leg was crippled but the child received no treatment. On inspection, there was some knock knee and the lower leg was bowed. The walk showed a very marked limp and sudden increase of the bow at each step. The weight was transmitted chiefly through the ligaments of the knee to the fibula and on weight bearing the give was so marked as to remind one of a congenital hip dislocation. It was easy to feel the nonunion and to move the fragments. There was shortening and atrophy. The X-ray showed the nonunion.

Fig. 2. X-rays showing fracture.

Operation at the Children's Hospital, September 18. Incision over the fracture and the ends cleared of dense fibrous tissue. The long end of the upper fragment was cut off and removed. Owing to muscular retraction the upper fragment could not be approximated to the lower in the extended position—only in a flexed position. Two canals were chiselled out above and below for a bone transplant. It was noted that the ends of the bone, especially the lower, were dense and hard and bled very little. Incision over the opposite tibia and graft of suitable size removed by electric saw. This was fitted in the right side and retained by kangaroo tendon and chromic catgut. The plaster cast had to be put on with the knee in considerable flexion to keep the fragments in line.

Fig. 3. X-rays showing bone splint.

In eight weeks the union was firm and walking was allowed after some time to strengthen the limb. Six months after this operation an osteotomy was done in the lower part of the same bone and the lower bow leg corrected. The bone was small in calibre but of good quality. Three months later, the second X-Rays, showing the graft and the site of the osteotomy, were taken, and the second photograph showing a straight leg. The sole of the shoe was increased to make up for the shortening.

## Figures and Tables

**Fig. 1. f1:**
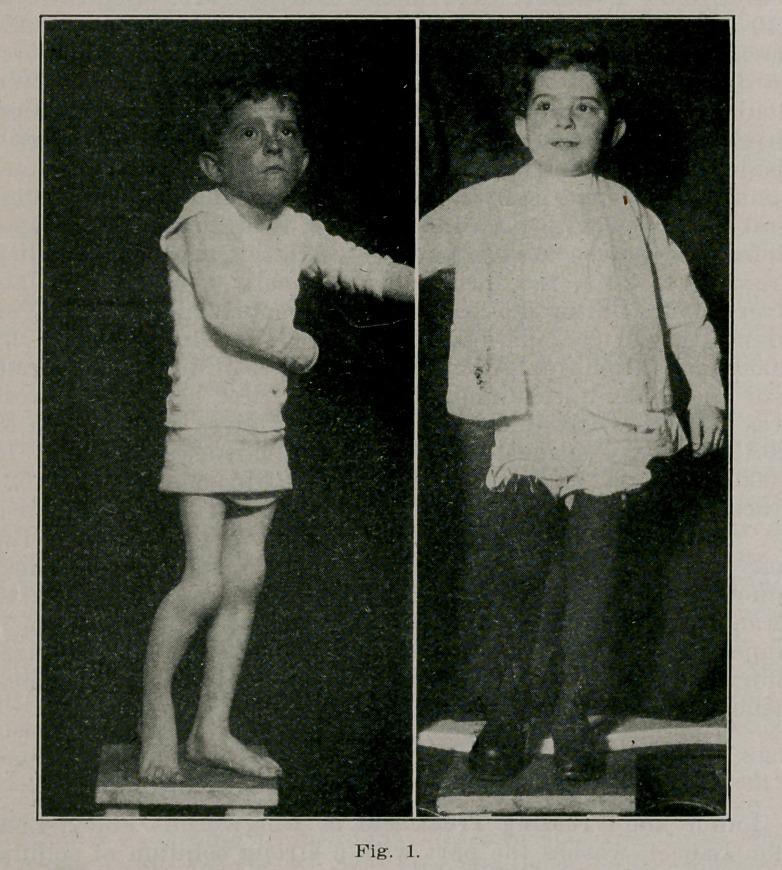


**Fig. 2. f2:**
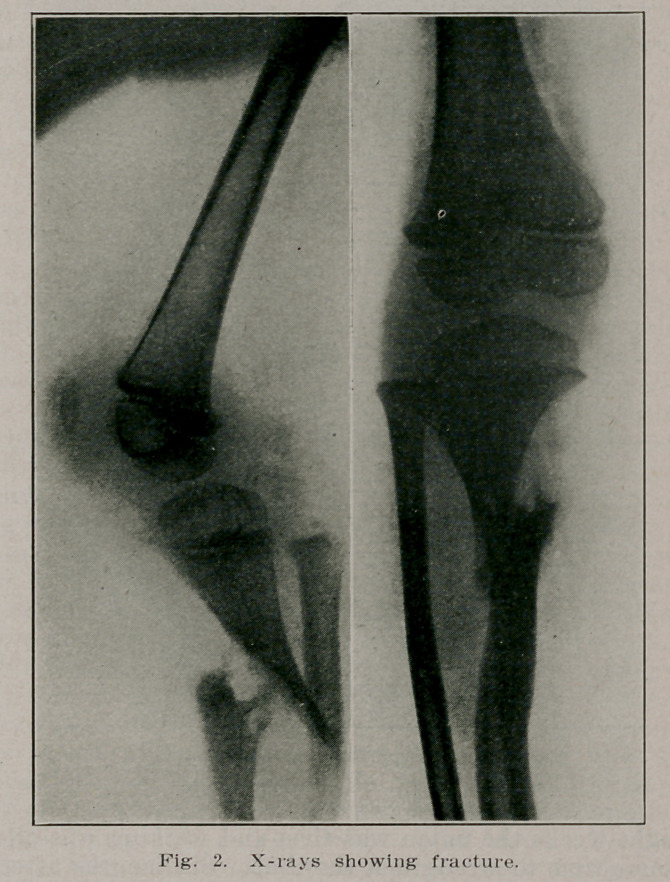


**Fig. 3. f3:**